# Supplementary material for: Adaptive evolution of an essential telomere protein restricts telomeric retrotransposons
Source: eLife. 2020 Dec 22;9:e60987. doi: 10.7554/eLife.60987 (PMC7755394; doi:10.7554/eLife.60987)
Supplement: Transparent reporting form [file elife-60987-transrepform.pdf]

eLIFE
1st Floor
24 Hills Road
Cambridge CB2 1JP, UK
P 01223 855340
W elifesciences.org
T @elife

eLife's transparent reporting form

We encourage authors to provide detailed information *within their submission* to facilitate the interpretation and replication of experiments. Authors can upload supporting documentation to indicate the use of appropriate reporting guidelines for health-related research (see EQUATOR Network), life science research (see the BioSharing Information Resource), or the ARRIVE guidelines for reporting work involving animal research. Where applicable, authors should refer to any relevant reporting standards documents in this form.

If you have any questions, please consult our Journal Policies and/or contact us: editorial@elifesciences.org.

Sample-size estimation

- You should state whether an appropriate sample size was computed when the study was being designed
- You should state the statistical method of sample size computation and any required assumptions
- If no explicit power analysis was used, you should describe how you decided what sample (replicate) size (number) to use

Please outline where this information can be found within the submission (e.g., sections or figure legends), or explain why this information doesn't apply to your submission:

We determined sample size based on previous experience and diminishing returns. We consistently used at least three biological replicates – three separate pools of ovaries for each genotype (RNA-seq, piRNA seq), three populations per experimentally evolved genotype, or three - six individual larvae per genotype and > or = 10 measurements per individual for cytological quantification of polytene signal. For the fertility assay, each vial of male and female parents corresponds to a biological replicate (30 replicates per genotype).

Replicates

- You should report how often each experiment was performed
- You should include a definition of biological versus technical replication
- The data obtained should be provided and sufficient information should be provided to indicate the number of independent biological and/or technical replicates
- If you encountered any outliers, you should describe how these were handled
- Criteria for exclusion/inclusion of data should be clearly stated
- High-throughput sequence data should be uploaded before submission, with a private link for reviewers provided (these are available from both GEO and ArrayExpress)

Please outline where this information can be found within the submission (e.g., sections or figure legends), or explain why this information doesn't apply to your submission:

Experiments that spanned multiple days were performed on both genotypes to minimize coupling of technical noise with biological signal. Pools of ovaries from each genotype correspond to biological replicates in our RNA-seq and piRNA-seq experiments. Our cytological experiments were performed multiple times on separate biological material (at least three separate individuals). The biological replicate number for each experiment can be found in the relevant figure legend and in the uploaded source data. The technical replicate number (e.g., # of nuclei sampled from a given individual) can be found in the source data and methods. No outliers were removed from the data. All high-throughput sequence data has been uploaded to NCBI (and project number reported in the manuscript).

Statistical reporting

- Statistical analysis methods should be described and justified
- Raw data should be presented in figures whenever informative to do so (typically when N per group is less than 10)
- For each experiment, you should identify the statistical tests used, exact values of N, definitions of center, methods of multiple test correction, and dispersion and precision measures (e.g., mean, median, SD, SEM, confidence intervals; and, for the major substantive results, a measure of effect size (e.g., Pearson's r, Cohen's d)
- Report exact p-values wherever possible alongside the summary statistics and 95% confidence intervals. These should be reported for all key questions and not only when the p-value is less than 0.05.

Please outline where this information can be found within the submission (e.g., sections or figure legends), or explain why this information doesn't apply to your submission:

Statistical tests used through the manuscript are reported clearly in the Methods section. We used only non-parametric tests that assume non-Gaussian data and unequal variance across groups (in our case, genotypes). The figure legends and source data associated with each plot report biological replicate number. Raw datapoints are shown when N per group is less than 10. Multiple test correction for bioinformatic data are reported clearly in Methods section. Exact p-values are reported whenever possible. For the subset of statistical test for which the software only reports "p-value < or >", the exact p-value is not reported (e.g., MWU). We report the exact p-value for the viability assay, which was greater than 0.05.

(For large datasets, or papers with a very large number of statistical tests, you may upload a single table file with tests, Ns, etc., with reference to sections in the manuscript.)

Group allocation

- Indicate how samples were allocated into experimental groups (in the case of clinical studies, please specify allocation to treatment method); if randomization was used, please also state if restricted randomization was applied
- Indicate if masking was used during group allocation, data collection and/or data analysis

Please outline where this information can be found within the submission (e.g., sections or figure legends), or explain why this information doesn't apply to your submission:

Experimental groups for all analyses are simply the genotypes ("HOAP[mel]" and "HOAP[yak]") that we engineered at the inception of the project.

Additional data files ("source data")

- We encourage you to upload relevant additional data files, such as numerical data that are represented as a graph in a figure, or as a summary table
- Where provided, these should be in the most useful format, and they can be uploaded as "Source data" files linked to a main figure or table
- Include model definition files including the full list of parameters used
- Include code used for data analysis (e.g., R, MatLab)
- Avoid stating that data files are "available upon request"

Please indicate the figures or tables for which source data files have been provided:

All data plotted/reported in the manuscript have a corresponding source data file with raw observations.
